# Alcohol intake and mortality among women with invasive breast cancer

**DOI:** 10.1038/bjc.2011.561

**Published:** 2012-01-03

**Authors:** H R Harris, L Bergkvist, A Wolk

**Affiliations:** 1 Division of Nutritional Epidemiology, Institute for Environmental Medicine, Karolinska Institutet, Box 210, 171 77, Stockholm, Sweden; 2 Obstetrics and Gynecology Epidemiology Center, Brigham and Women's Hospital, Harvard Medical School, 221 Longwood Avenue, Boston, MA 02115, USA; 3 Department of Surgery and Centre for Clinical Research, Central Hospital, 721 89 Västerås, Sweden

**Keywords:** alcohol, breast cancer, survival

## Abstract

**Background:**

Alcohol intake has consistently been associated with increased breast cancer incidence in epidemiological studies. However, the relation between alcohol and survival after breast cancer diagnosis is less clear.

**Methods:**

We investigated whether alcohol intake was associated with survival among 3146 women diagnosed with invasive breast cancer in the Swedish Mammography Cohort. Alcohol consumption was estimated using a food frequency questionnaire. Cox proportional hazard models were used to calculate hazard ratios (HRs) and 95% confidence intervals (95% CIs).

**Results:**

From 1987 to 2008 there were 385 breast cancer-specific deaths and 860 total deaths. No significant association was observed between alcohol intake and breast cancer-specific survival. Women who consumed 10 g per day (corresponding to approximately 0.75 to 1 drinks) or more of alcohol had an adjusted HR (95% CI) of breast cancer-specific death of 1.36 (0.82–2.26;p_trend_=0.47) compared with non-drinkers. A significant inverse association was observed between alcohol and non-breast cancer deaths. Those who consumed 3.4–9.9 g per day of alcohol had a 33% lower risk of death compared with non-drinkers (95% CI 0.50–0.90;p_trend_=0.04).

**Conclusion:**

Our findings suggest that alcohol intake up to approximately one small drink per day does not negatively impact breast cancer-specific survival and a half drink per day is associated with a decreased risk of mortality from other causes.

Alcohol intake has consistently been associated with increased breast cancer risk in epidemiological studies. (Smith-Warner *et al*, 1998; AICR, 2007) In contrast to the increased risk observed with breast cancer incidence, most studies examining survival after breast cancer diagnosis have either reported an inverse association between pre- and post-diagnosis alcohol consumption and all-cause survival (Barnett *et al*, 2008; Reding *et al*, 2008; Beasley *et al*, 2010; Flatt *et al*, 2010) or no association. (Ewertz *et al*, 1991; Zhang *et al*, 1995; Holmes *et al*, 1999; Saxe *et al*, 1999; McEligot *et al*, 2006; Franceschi *et al*, 2009; Kwan *et al*, 2010). When breast cancer-specific outcomes have been examined, four studies have reported an increased risk with breast cancer-specific mortality or recurrence (Hebert *et al*, 1998; Green McDonald *et al*, 2002; Beasley *et al*, 2010; Kwan *et al*, 2010), and two studies reported no association (Rohan *et al*, 1993; Goodwin *et al*, 2003). Given these inconclusive results, the aim of this study was to investigate whether pre-diagnosis alcohol intake was associated with breast cancer-specific and overall survival among women with invasive breast cancer in the population-based Swedish Mammography Cohort (SMC). We also examined whether the association between alcohol and survival differed by hormone receptor status, disease stage at diagnosis, or folate intake.

## Materials and methods

### Study population

This study included 3146 participants in the SMC with invasive breast cancer diagnosed between 1987 and 2008. Recruitment and characteristics of this cohort have been previously described (Wolk *et al*, 2006). In brief, the SMC is a population-based cohort of 66 651 women born between 1914 and 1948 that were recruited between 1987 and 1990 in Västmanland and Uppsala counties in central Sweden. A total of 66 651 women (74% of the source population) returned the baseline questionnaire with questions regarding diet, reproductive factors, body size, and other factors. In 1997, a second questionnaire was sent to 56 030 participants who were still alive and residing in the study area; 39 227 (70%) women returned this questionnaire. Those with an incorrect or missing national registration number, previous cancer diagnosis (except non-melanoma skin cancer), and with implausible total energy intake (three standard deviations (s.d.) from the mean value for log_e_-transformed energy intake) were excluded from the baseline cohort. The study was approved by the ethics committees at the Uppsala University Hospital (Uppsala, Sweden) and the Karolinska Institutet (Stockholm, Sweden).

Histologically confirmed incident invasive breast cancer cases were ascertained by linkage of the study cohort with Swedish Cancer registers. Estrogen (ER) and progesterone (PR) receptor status and other clinical characteristics were obtained by reviewing pathology laboratory works logs from Uppsala University Hospital (1987–1994) and by linkage with the clinical database (Quality Register) at the Regional Oncology Centre in Uppsala (1992–2008), which was based on the patient's original medical records. ER and PR status, menopausal status at diagnosis, tumor size, grade, lymph node involvement, and type of treatment were available for approximately 77% of the cases. More detailed information on the evaluation of ER and PR status in this cohort has been described previously (Larsson *et al*, 2009).

### Dietary assessment

Diet was assessed using a 67-item food frequency questionnaire (FFQ) at baseline and a 96-item FFQ in 1997. Participants were asked how often, on average, they had consumed alcohol during the previous 6 months (1987) or year (1997). Eight responses were possible ranging from never or seldom to four, or more times per day. Alcohol intake was calculated by multiplying the reported frequency of consumption by age-specific drinks sizes (Suzuki *et al*, 2005). The FFQ has been previously validated among 129 cohort participants and the correlation coefficient between the questionnaire and four 1-week dietary records was 0.9 for alcohol (A Wolk, 1992, unpublished data).

### Outcome assessment

Date of death was identified through linkage of the study cohort to the Swedish National Death Registry at Statistics Sweden. Cause of death was determined by International Classification of Diseases (ICD) codes (ICD9 and ICD10) through linkage to the Cause of Death Registry at the National Board of Health and Welfare.

### Statistical analysis

We used Cox proportional hazard models with time since diagnosis in months as the time scale to calculate hazard ratios (HRs) and 95% confidence intervals (95% CIs) for death from breast cancer (primary endpoint), death from other causes, or death from any cause as the endpoints, depending on the analysis. Participants contributed person-time from the date of breast cancer diagnosis until the death from breast cancer, death from another cause, or end of follow-up on 31 December 2008. Baseline diet (1987) was considered the exposure in all analyses except when dietary change was examined. Alcohol consumption was divided into four categories: non-drinker (reference), <3.4, 3.4–9.9, and ⩾10 g per day. The cut points of 3.4 and 10 g per day correspond to the median value among drinkers in the full SMC and to approximately one alcoholic drink, respectively (Suzuki *et al*, 2005). Total caloric intake (continuous) and age at diagnosis (continuous) were included in all models.

Models were adjusted *a priori* for total caloric intake, age at diagnosis, education level (primary school, high school, university), marital status (single, married, divorced, widowed, living with partner), menopausal status at diagnosis (premenopausal, postmenopausal, unknown), body mass index (BMI) (<20, 20–24.9, 25–29.9, ⩾30 kg m^−2^), and calendar year of diagnosis (continuous). We considered parity, age at first birth, oral contraceptive use, postmenopausal hormone use, height, and family history of breast cancer to be potential confounders if their addition to the model changed the HR by at least 10%. Based on these criteria they were not observed to be confounders and therefore were not included in the final models. Additional multivariable models considered adjustment for clinical characteristics: stage (I, II, III, IV), grade (I, II, III), radiation treatment (yes/no), and chemotherapy/hormonal therapy (no chemotherapy or hormonal therapy, hormonal therapy and no chemotherapy, chemotherapy and no hormonal therapy, hormonal therapy and chemotherapy).

We examined whether the association between alcohol and breast cancer survival differed by hormone receptor status, disease stage at diagnosis, or folate intake with a likelihood ratio test comparing the model with the cross-product term between alcohol and each potential effect modifier to the model with main effects only. All tests of statistical significance were two-sided, and all statistical analyses were performed using SAS Version 9.2 (SAS Institute Inc., Cary, NC, USA).

## Results

During 25 940 person-years of follow-up from 1987 to 2008 there were 860 total deaths and 385 breast cancer deaths among 3146 breast cancer cases. Thirty percent of participants were non-drinkers and the median alcohol intake among the drinkers was 3.0 g per day. The distribution of participant characteristics by alcohol intake are shown in Table 1. Women with the highest intake of alcohol were more likely to have a postsecondary education and to be married, were less often nulliparous, and had the lowest mean BMI. Of those with ER and PR status available 62% were ER+/PR+, 20% were ER+/PR−, 5% were ER−/PR+, and 13% ER−/PR−.

No significant association was observed between alcohol intake and breast cancer-specific mortality (Table 2). Women who consumed 10 or more g per day of alcohol had a covariate-adjusted HR (95% CI) of breast cancer death of 1.09 (0.66–1.81) compared with non-drinkers (p_trend_=0.87) and a covariate and clinical-characteristics adjusted HR (95% CI) of 1.36 (0.82–2.26; p_trend_=0.47). When death from a cause other than breast cancer was considered the endpoint there was a statistically significant decrease in risk that was strongest among moderate drinkers. Those who consumed 3.4–9.9 g per day of alcohol had a 33% lower risk of non-breast cancer-related death compared with non-drinkers (95% CI 0.50–0.90; p_trend_=0.04). This reduction in risk was not statistically significant for women consuming 10 or more g per day of alcohol; however, there were few women in this subgroup (HR=0.81; 95% CI 0.46–1.43). The association between alcohol intake and mortality did not vary by the hormone receptor status of the tumor, disease stage at diagnosis or folate intake (data not shown). When analyses were restricted to postmenopausal women at diagnosis results were similar to the full analytic sample.

We examined dietary change following breast cancer diagnosis among the 717 breast cancer cases who were diagnosed with breast cancer from 1987–1996 and completed a FFQ in 1997 after their breast cancer diagnosis. Over 92% remained in the same or adjacent category of alcohol intake following their breast cancer diagnosis. However, numbers were too small to examine the association between post-diagnosis alcohol intake and survival in this group.

## Discussion

In this population-based prospective study of Swedish women with breast cancer, alcohol consumption was not significantly associated with breast cancer-specific mortality, whereas moderate intake was associated with a decreased risk of mortality from causes other than breast cancer.

Alcohol intake is well established as a risk factor for breast cancer occurrence (Smith-Warner *et al*, 1998; AICR, 2007). The biological mechanism through which it increases breast cancer risk is most likely through increases in ER levels (Dorgan *et al*, 2001; Singletary and Gapstur, 2001; Dumitrescu and Shields, 2005). Alcohol intake has the potential to influence survival through a similar mechanism as a number of breast cancer treatments include anti-ER therapy, and some studies have shown that hormone replacement therapy may increase breast cancer recurrence (Ewertz *et al*, 1991; Kenemans *et al*, 2009). However, studies examining alcohol intake and survival after breast cancer have not consistently demonstrated a similar unfavorable effect. This may be in part because they have used various endpoints to examine survival (Holmes, 2010). With all-cause mortality as the endpoint seven studies reported no association (Ewertz *et al*, 1991; Zhang *et al*, 1995; Holmes *et al*, 1999; Saxe *et al*, 1999; McEligot *et al*, 2006; Franceschi *et al*, 2009; Kwan *et al*, 2010) and four studies reported an inverse association between alcohol consumption and overall survival (Barnett *et al*, 2008; Reding *et al*, 2008; Beasley *et al*, 2010; Flatt *et al*, 2010). In contrast, when the endpoint was breast cancer-specific mortality or breast cancer recurrence, two studies reported no association (Rohan *et al*, 1993; Goodwin *et al*, 2003), while four studies reported an increase in risk with increasing alcohol consumption (Hebert *et al*, 1998; Green McDonald *et al*, 2002; Beasley *et al*, 2010; Kwan *et al*, 2010). We observed no significant association between alcohol intake and breast cancer-specific mortality, although there was the suggestion of an increased risk among the small number of women consuming 10 g per day (corresponding to approximately 0.75 to 1 drink/day) or more (HR=1.36; 95% CI 0.82–2.26) (Suzuki *et al*, 2005).

In contrast to our results for breast cancer-specific mortality, we observed a decrease in risk for mortality from causes other than breast cancer that was strongest for moderate drinkers (those consuming on average approximately a half drink per day). These results are consistent with the observation that while moderate alcohol intake reduces the risk of cardiovascular disease mortality, heavier drinking may increase the risk of death from breast cancer (Fuchs *et al*, 1995).

Our study was limited by a single pre-diagnosis assessment of diet in the majority of our participants, which did not allow for us to examine post-diagnosis diet during the follow-up period. However, when we examined dietary change in the subset of breast cancer cases who completed a FFQ post-diagnosis, over 92% remained in the same or adjacent category of alcohol intake following diagnosis. In addition, measurement error was possible in our assessment of dietary intake as it was collected via a self-administered FFQ and alcohol portion size was not assessed on the 1987 FFQ. However, the FFQ has been previously validated with diet records with a correlation of 0.9 for alcohol (A Wolk, unpublished data, 1992) and any errors in this measurement would likely cause an attenuation of the true effect. Another limitation of our study was the low consumption of alcohol in our population, which may have limited our ability to detect a significant association between higher levels of alcohol intake and breast cancer-specific mortality. The mean alcohol intake in the SMC is 3.2 g per day, which is much lower than other prospective cohorts consisting of healthy women at baseline that have reported means ranging from 5.5–12.6 g per day (Smith-Warner *et al*, 1998). Finally, we only had ER and PR status, menopausal status at diagnosis, tumor size, grade, lymph node involvement, and type of treatment information for 77% of our cases.

Our study has several strengths including use of breast cancer cases diagnosed within a prospective population-based cohort, complete follow-up of all cases, and a relatively large number of total deaths (*n*=860), including 385 breast cancer deaths. This large sample size allowed us the power to examine breast cancer-specific mortality as well as how the association differed by hormone receptor status and stage at diagnosis.

In conclusion, our findings suggest that alcohol intake at the level of up to approximately one small drink per day does not negatively impact breast cancer-specific survival and is inversely associated with non-breast cancer death. These results are consistent with the World Cancer Research Fund/American Institute for Cancer Research recommendation that if alcohol drinks are consumed at all, alcohol consumption should be limited to no more than one small drink a day for women (AICR, 2007). The association between alcohol intake and breast cancer specific-mortality should be further evaluated in populations with a wider range of alcohol consumption.

## Figures and Tables

**Table 1:** Characteristics of 3146 women with invasive breast cancer in the Swedish Mammography Cohort by alcohol intake^a^

	**Alcohol intake (g per day)**			
	**Non-drinker**	**<3.4**	**3.4–9.9**	**⩾10**
Median alcohol intake (g per day)	0	1.8	5.0	12.4
Age at enrollment (years)	56.8	53.0	49.9	48.4
Age at diagnosis (years)	68.2	64.9	62.6	61.4
Postsecondary education (%)	8.3%	12.4%	14.2%	18.2%
Married (%)	68.1%	69.8%	71.9%	82.8%
Body mass index (kg m^−2^)	26.3	24.9	23.9	23.6
Height (cm)	163.5	164.8	165.3	165.8
Age at menarche (years)	13.3	13.2	13.1	13.3
Nulliparous (%)	14.4%	13.2%	11.4%	10.9%
Age at first birth among parous women (years)	24.6	24.5	24.8	25.1
Number of children	2.4	2.2	2.2	2.1
Family history of breast cancer (%)	13.2%	10.8%	11.2%	9.7%
Ever use of oral contraceptives (%)	35.2%	43.4%	51.5%	56.9%
Postmenopausal at diagnosis (%)^b^	93.9%	89.7%	88.6%	89.7%
Total energy intake (kcal per day)	1575	1571	1610	1620
Stage I (%)	48.5%	52.4%	53.4%	60.3%
				
*Treatment (%)* b ^,^ c				
Radiation	47.3%	58.4%	62.5%	63.6%
Chemotherapy	10.5%	16.2%	18.4%	19.4%
Hormonal	33.2%	34.4%	37.5%	37.0%
				
Estrogen receptor positive (%)^b^	81.2%	81.3%	82.5%	86.1%
Progesterone receptor positive (%)^b^	69.5%	68.4%	63.9%	61.7%

a Data represent mean unless otherwise indicated. Percents may not equal 100 due to missing values.

b Menopausal status at diagnosis, ER and PR status, and type of treatment were available for 77% of the cases.

c Greater than 100% because some breast cancer patients receive more than one treatment.

**Table 2:** Hazard ratios (HR) and 95% confidence intervals (95% CI) of death by categories of alcohol intake among 3146 invasive breast cancer cases in the Swedish Mammography Cohort

	**Alcohol intake (g per day)**				
	**Non-drinker**	**<3.4**	**3.4–9.9**	**⩾10**	***P*_trend_** a
Median alcohol intake (g per day)	0	1.8	5.0	12.4	
Person-years	7714	10 431	6515	1280	
					
*Breast cancer deaths*	*133*	*149*	*85*	*18*	
Age-adjusted model	1.00	0.85 (0.68–1.08)	0.79 (0.60–1.05)	0.85 (0.51–1.39)	0.26
Covariate-adjusted model^b^	1.00	0.93 (0.73–1.18)	0.94 (0.70–1.25)	1.09 (0.66–1.81)	0.87
Covariate-adjusted model+clinical characteristics^c^	1.00	0.95 (0.74–1.21)	0.94 (0.70–1.25)	1.36 (0.82–2.26)	0.47
					
*Non-breast cancer deaths*	*224*	*172*	*66*	*13*	
Age-adjusted model	1.00	0.74 (0.61–0.91)	0.65 (0.49–0.85)	0.77 (0.44–1.35)	0.01
Covariate-adjusted model^b^	1.00	0.76 (0.62–0.93)	0.66 (0.50–0.89)	0.79 (0.45–1.29)	0.03
Covariate-adjusted model+clinical characteristics^c^	1.00	0.77 (0.73–0.95)	0.67 (0.50–0.90)	0.81 (0.46–1.43)	0.04
					
*Total deaths*	*357*	*321*	*151*	*31*	
Age-adjusted model	1.00	0.79 (0.68–0.92)	0.71 (0.59–0.87)	0.80 (0.56–1.16)	0.01
Covariate-adjusted model^b^	1.00	0.83 (0.71–0.97)	0.79 (0.64–0.96)	0.92 (0.63–1.34)	0.16
Covariate-adjusted model+clinical characteristics^c^	1.00	0.85 (0.73–1.00)	0.80 (0.65–0.98)	1.03 (0.71–1.51)	0.36

a Determined using category medians.

b Cox proportional hazard model adjusted for age (continuous), energy intake (continuous), education level (primary, high school, university), marital status (single, married, divorced, widowed, living with partner), menopausal status at diagnosis (premenopausal, postmenopausal, unknown), body mass index (<20, 20–24.9, 25–29.9, ⩾30 kg m^−2^), and calendar year of diagnosis (continuous).

c Cox proportional hazard model adjusted for the variables above plus disease stage (I, II, III/IV), grade (I, II, III), radiation treatment (yes/no), and chemotherapy and/or hormonal treatment (no chemotherapy or hormonal treatment, hormonal therapy and no chemotherapy, chemotherapy and no hormonal therapy, and hormonal therapy and chemotherapy).
